# Solidification Performance and Mechanism of TSC Composite Soil Based on Microbially Induced Mineralization

**DOI:** 10.3390/ma19091775

**Published:** 2026-04-27

**Authors:** Haowei Ding, Qiwei Zhan, Haitao Hu, Yiming Xiong

**Affiliations:** 1 School of Civil Engineering and Architecture, Jiangsu University of Science and Technology, Zhenjiang 212000, China; 241710901101@stu.just.edu.cn (H.D.); 231110901120@stu.just.edu.cn (Y.X.); 2 Zhenjiang Key Laboratory of Infrastructure Renewal and Resilience Enhancement, Zhenjiang 212000, China; 3 School of Transportation, Southeast University, Nanjing 211189, China; 23025939@seu.edu.cn

**Keywords:** TSC composite soil, microbial-induced mineralization, soil stabilization, water stability, microstructure

## Abstract

To enhance the engineering performance of fine-grained composite soils with unbalanced particle gradation, high plasticity, and poor water stability, a synergistic stabilization strategy combining particle structure regulation and microbially induced calcium carbonate precipitation (MICP) was proposed. The particle size distribution and fundamental engineering properties of a titanium gypsum–clay (TSC) composite soil were first optimized through systematic single-factor blending tests. The results indicate that a TS:C ratio of 60:40 significantly improved gradation characteristics, reduced plasticity, and enhanced both compaction behavior and load-bearing capacity. Based on the optimized gradation framework, MICP treatment was subsequently introduced to further enhance water stability. The effects of key parameters, particularly the type of calcium source, on the evolution of water stability were systematically investigated. X-ray diffraction (XRD) and scanning electron microscopy (SEM) were employed to elucidate the underlying reinforcement mechanisms. The results demonstrate that the water stability coefficient increased markedly from 0.35 to 0.83 following MICP treatment, while strength degradation under water immersion was effectively mitigated. Microscopic observations reveal that microbially precipitated calcite fills pore spaces and forms a continuous cementation network via particle bridging and interfacial bonding, leading to an approximately 32% reduction in porosity. Overall, the proposed synergistic strategy offers an effective and sustainable approach for improving the water stability and structural integrity of complex fine-grained composite soils.

## 1. Introduction

Fine-grained composite soils with complex mineral compositions and unbalanced particle gradation are commonly encountered in engineered soil blending and land reuse applications [1]. However, such soils typically exhibit inherent deficiencies, including high plasticity, strong water sensitivity, and poor water stability [2], which result in structural softening and strength degradation under water immersion or prolonged moist conditions [3]. These unfavorable characteristics significantly limit their applicability in geotechnical engineering. Conventional stabilization methods, such as cement and lime treatment, can effectively improve soil strength in the short term; however [4], their drawbacks have become increasingly evident, including high carbon emissions, hydration heat-induced brittle cracking [5], and progressive performance deterioration under prolonged water exposure. Physical modification approaches, such as crushing and sieving to optimize particle gradation, can partially improve basic engineering properties but are generally insufficient to fundamentally address the issue of poor water stability [6]. In addition, emerging polymer-based chemical stabilization methods exhibit certain effectiveness; however, they are often constrained by high costs and potential risks of secondary environmental pollution [7]. Consequently, the development of stabilization techniques that simultaneously enhance mechanical performance, ensure economic feasibility, and maintain environmental sustainability has become a critical challenge in the field of fine-grained soil stabilization.

In recent years, microbial-induced calcium carbonate precipitation (MICP) [8] has attracted considerable attention due to its low energy consumption, low carbon footprint, and controllable mineralization process. Through in situ biomineralization, MICP promotes interparticle cementation and pore filling, thereby improving soil structural integrity and resistance to water-induced damage [9]. Nevertheless, the effectiveness of MICP and the associated mechanisms governing water stability evolution in fine-grained composite soils with complex mineralogy and high impurity contents remain insufficiently understood [10]. In addition to MICP, enzyme-induced carbonate precipitation (EICP) has also been considered a promising bio-mediated ground improvement technique. Unlike MICP, which relies on living ureolytic microorganisms, EICP uses free urease to catalyze urea hydrolysis. This may simplify implementation in some cases, but MICP has advantages in terms of bacterial surface-mediated nucleation, sustained microbial activity, and stronger interparticle bonding within soil matrices [11]. Meanwhile, the long-term reliance on natural clay resources for composite soil construction has intensified regional pressures on soil resource exploitation [12]. In recent years, numerical modeling has also been increasingly applied to the study of fine-grained composite soils with complex mineral compositions. These models typically consider key structural parameters such as particle size distribution, pore structure characteristics, interparticle bonding strength, and mineral composition to simulate the mechanical behavior and stability of the material. In particular, parameters related to porosity, contact mechanics, and cementation evolution are critical for capturing the relationship between microstructure and macroscopic performance. From a sustainability perspective, the use of industrial by-products such as titanium gypsum [13] as a partial replacement for natural clay not only reduces the consumption of primary soil resources, but also creates new opportunities for particle structure regulation and performance optimization of fine-grained composite soils.

Extensive research has been conducted both domestically and internationally; however, significant limitations persist. In terms of physical blending, F. Zha et al. [14] demonstrated that titanium gypsum can improve the stabilization performance of expansive soil by increasing the proportion of coarse particles, thereby enhancing soil strength. Nevertheless, the long-term water stability and durability of this approach remain unverified, and its technical reliability requires further assessment. A. Parhizkar et al. [15] applied a synergistic modification of gypsum soil using a slag–lime–fly ash combination, achieving maximum strength at 28 days; however, strength decreased at 56 days due to excessive hydration of calcium aluminate hydrates, and the mixture formulation was complex. In cement blending, G. Wang et al. [16] reported that titanium gypsum, at an appropriate proportion, could function as a cement retarder; however, the low blending ratio of approximately 5% limited the scalability of this approach. In microbial modification, H. Bao et al. [17] increased phosphogypsum strength by 40% using Bacillus paracasei; however, efficiency remained low in the absence of physical blending. Y. Wang et al. [18] substantially enhanced soft soil strength via biochar–MICP synergistic reinforcement; however, this method did not address titanium gypsum systems and lacked mechanistic evidence in high-impurity environments. In summary, existing research exhibits two primary gaps: first, single modification technologies struggle to simultaneously ensure strength, water stability, and cost-effectiveness; second, the application mechanism of MICP in high-impurity titanium gypsum environments remains unclear, and the transferability of analogous calcium-based solid waste technologies is limited [19].

To address these gaps, this study establishes a core framework of “targeted regulation of fundamental properties followed by high-efficiency enhancement of water stability” and performs three key investigations. First, clay blending experiments were conducted to evaluate parameters such as particle size distribution and liquid/plastic limits at varying blending ratios, aiming to determine the optimal proportion. Second, microbial-induced calcium carbonate precipitation (MICP) reinforcement experiments were performed using a controlled-variable approach to investigate the effects of calcium source type, bacterial suspension dosage, curing temperature, and pH [20] on the water stability coefficient. Finally, characterization techniques—including X-ray diffraction (XRD), Fourier transform infrared spectroscopy (FTIR), and scanning electron microscopy (SEM)—were applied to elucidate the “filling–bonding” enhancement mechanism. The study clarifies the synergistic mechanism between clay blending and MICP, highlighting MICP’s nucleation selectivity and pore-filling effects in high-impurity environments. This mechanistic analysis not only addresses a theoretical gap in understanding MICP-induced mineralization in high-impurity calcium-based solid wastes, but also provides a foundation for investigating the mechanisms of composite modification technologies.

## 2. Materials and Methods

### 2.1. Raw Materials

#### 2.1.1. Analysis of Soil Material

**Figure 1:**
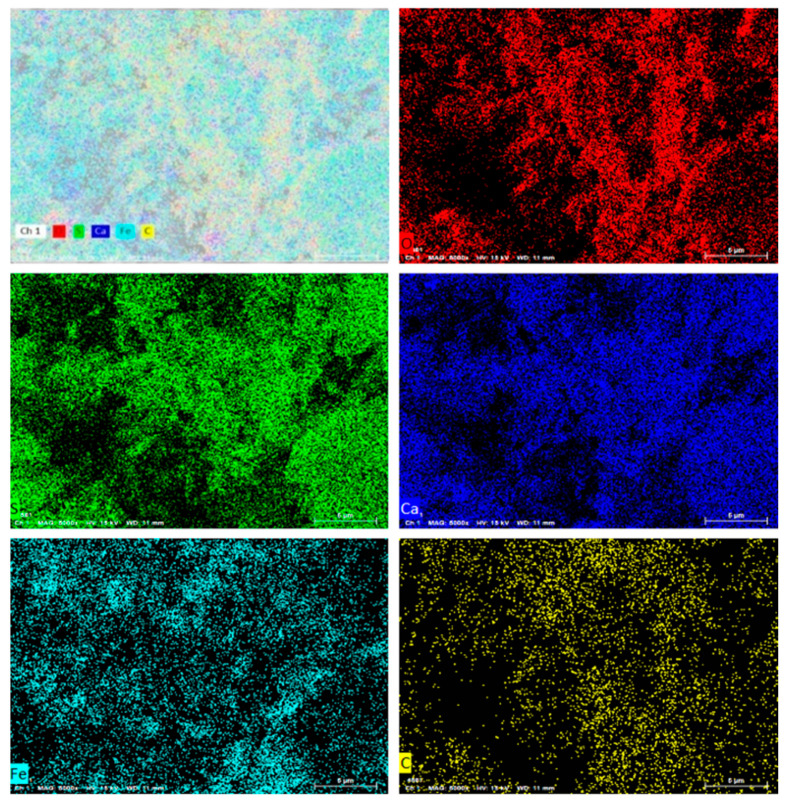
Titanium Gypsum Energy Spectrum.

Titanium gypsum (TS) and clay were selected as the raw materials for this study. The titanium gypsum was obtained from an industrial byproduct of a sulfuric acid-based titanium dioxide plant in Zhenjiang, Jiangsu Province, and served as the primary research material. The clay was collected from soft riverbank soil in the Zhenjiang region. X-ray fluorescence spectroscopy (XRF) [21] was employed to determine the chemical composition of the raw materials, and the results are presented in Table 1. As shown in Table 1, titanium gypsum mainly consists of SO_3_, CaO, Fe_2_O_3_, and SiO_2_, whereas clay primarily comprises SiO_2_, Al_2_O_3_, and Fe_2_O_3_, with SiO_2_ being the dominant component at 62.36%. To confirm the elemental composition of titanium gypsum, additional testing was performed using an energy-dispersive spectrometer, and the results are shown in Figure 1. The results indicate that titanium gypsum primarily contains C, O, S, and Ca, which is consistent with the XRF analysis and confirms the reliability of the compositional assessment. After air-drying, the basic physical properties of the raw materials—including pH, liquid limit, plastic limit, dry density, and free expansion rate—were measured, and the results are listed in Table 2. The measurements reveal significant differences in fundamental physical properties between titanium gypsum and clay. Titanium gypsum exhibits a pH of 9.46, markedly higher than clay’s 8.02, indicating strong alkalinity. The liquid and plastic limits of titanium gypsum (67.82% and 30.96%, respectively) are substantially higher than those of clay, indicating higher water absorption and plasticity. These properties predispose titanium gypsum to strength degradation and volumetric deformation under humid conditions, thereby compromising engineering stability. The dry density of titanium gypsum is only 1128.79 kg·m^−3^, considerably lower than clay’s 1582.10 kg·m^−3^, reflecting its loose particle structure and high porosity, which hinder the formation of a stable framework. Its free expansion rate reaches 42%, about 1.6 times that of clay, further confirming its sensitivity to expansion and poor volumetric stability in water-rich environments. Overall, titanium gypsum exhibits unfavorable engineering properties—including high liquid limit, high free expansion rate, and low dry density—failing to satisfy the dual requirements of strength and water stability for road subgrade fill materials. Therefore, modification techniques such as clay blending are necessary to regulate particle gradation, reduce liquid plasticity, and improve structural compactness, providing a practical basis for optimizing the engineering performance of titanium gypsum.

#### 2.1.2. Cultivation of Mineralizing Strains and Preparation of Binding Solution

The urease-producing microorganism used in this study was *Bacillus pasteurii* (ATCC11859), supplied as a glycerol-preserved strain by Beijing Baozang Biotechnology Co., Ltd. (Beijing, China). *Bacillus pasteurii* synthesizes large quantities of urease during cellular metabolism, with urease content reaching approximately 1% of the dry cell weight. During the experiment, carbonate ions generated via urease-mediated urea hydrolysis reacted with calcium ions in the cementation solution to induce calcium carbonate precipitation. The strain was cultured in 150 mL of liquid medium containing 15 g/L peptone, 5 g/L yeast extract, 5 g/L potassium dihydrogen phosphate, and ultrapure water up to 1 L. The prepared medium was sterilized by autoclaving at 121 °C for 20 min. After cooling to room temperature, *Bacillus pasteurii* was inoculated at 1% (*v*/*v*) and incubated at 30 °C in a shaking incubator at 230 rpm. The cementation solution consisted of urea and a calcium source. In this study, the molar ratio of urea to Ca^2+^ was controlled at 1:1 to provide sufficient carbonate generation and calcium availability for microbial-induced calcium carbonate precipitation (MICP). In the MICP process, urease secreted by the bacteria catalyzes the hydrolysis of urea, producing NH^4+^ and CO_3_^2−^/HCO^3−^, while the increase in pH promotes carbonate formation. The generated carbonate ions then react with Ca^2+^ to form CaCO_3_ precipitates, which contribute to pore filling and particle bonding [22]. All subsequent MICP experiments, including the investigations of calcium source, pH, temperature, and bacterial suspension dosage, were conducted using this strain.

### 2.2. Test Plan

This study established a two-stage experimental framework of “clay compounding regulation—MICP reinforcement” to achieve synergistic enhancement of the road performance of titanium gypsum materials. In the first stage, clay content was used as the primary adjustment parameter, and systematic compound modification tests were conducted to meet fundamental engineering performance requirements. Key indicators—including particle gradation, liquid limit, compaction characteristics, California bearing ratio, and free expansion rate—were evaluated to determine the optimal clay dosage capable of achieving coordinated improvement across multiple basic properties. Based on this optimized compounding system, the second stage implemented MICP reinforcement experiments to further enhance material water stability. The influence patterns of core reaction parameters—such as calcium source type, bacterial solution dosage, curing temperature, and pH—were investigated to establish the optimal mineralization conditions for the titanium gypsum system. Subsequently, microstructural characterization techniques were applied to elucidate the synergistic enhancement mechanism between clay blending and MICP reinforcement from the perspectives of mineral phase evolution and pore structure reconstruction, thereby providing macro-to-micro theoretical support for the observed performance improvements.

#### 2.2.1. Composite Modification Experiments

To systematically evaluate the modification effects of the titanium gypsum–clay composite (TSC) on material properties, this study adopted a single-factor controlled variable approach. Clay content was selected as the core variable and configured at five gradient levels: 10%, 20%, 30%, 40%, and 50%. To avoid interference from extraneous factors, all test groups used raw materials from the same batch and strictly followed identical pretreatment procedures to ensure consistent experimental baselines. The specific group settings and mixing ratios are presented in Table 3. For core road performance evaluation, a series of tests and comparative analyses were performed on specimens from each group. The measured indicators included particle size distribution, liquid limit (W_L_), plastic limit (W_P_), optimum moisture content (OMC), maximum dry density (MDD), California bearing ratio (CBR), and free expansion rate. Water stability was primarily assessed using the strength retention rate. No fewer than three parallel specimens were tested for each indicator to ensure data reliability. The test results are statistically reported as “mean ± standard deviation,” and trends in performance variations with clay dosage were systematically analyzed. This process ultimately determined the optimal compounding ratio that balances performance and workability. Pure titanium gypsum was used as the baseline control group to clearly demonstrate the modification effects through comparative analysis.

#### 2.2.2. Microbial Curing Test

To evaluate the reinforcement effect of MICP on titanium gypsum–clay composites, four key process parameters were investigated: calcium source, pH, temperature, and bacterial suspension dosage. For calcium source selection, widely used and cost-effective calcium chloride was used as the benchmark, while calcium nitrate, calcium acetate, and calcium lactate served as comparison groups. All four calcium sources are highly water-soluble but differ in anion type, allowing analysis of how anion variation affects *Bacillus pasteurii* activity, calcium carbonate crystal morphology, and cementation capacity [23]. Four pH levels (6, 7, 8, and 9) were set, covering both the optimal growth and urease activity intervals of *Bacillus pasteurii* and typical pH fluctuations in engineering soils, facilitating assessment of how acid–base conditions influence mineralization reaction characteristics [24]. Four temperature levels (10, 20, 30, and 40 °C) were applied. Although 30 °C is the optimal growth temperature for *Bacillus pasteurii*, the 10–40 °C range simulates diverse construction environments, allowing evaluation of MICP applicability under varying climatic conditions [25]. Bacterial solution dosage was set at 0%, 10%, 20%, 30%, 40%, and 50% by volume, with 0% acting as a blank control to eliminate interference from external variables. The 10–50% dosage range reflects the positive correlation between bacterial concentration and urease activity while avoiding potential issues such as excessive costs and reduced system permeability. This ensures a comprehensive balance among mineralization efficiency, economic feasibility, and engineering applicability [26]. To maintain scientific rigor and comparability, all other experimental conditions were kept constant except for the variables under investigation. These controlled conditions included the molar ratio of urea to calcium source, binder dosage, compaction method, and curing temperature and humidity. The detailed experimental procedures are described below.

(1) Urease-producing bacteria were cultured to the logarithmic growth phase and enriched, maintaining culture turbidity at OD_600_ > 1.5. The binder solution was prepared according to the experimental design.

(2) The base material TSC4 (titanium gypsum:clay = 60:40) was weighed and thoroughly mixed with the bacterial suspension at the specified dosage and moisture content following the protocol. The mixture was poured into a mold coated internally with petroleum jelly, compacted in layers by vibration, and leveled at the surface.

(3) Specimens were placed in a standard curing chamber and cured at the factor-specific temperature until the predetermined age was reached. After curing, specimens were demolded, surface moisture was removed, and mechanical and water stability tests were immediately conducted.

### 2.3. Test Methods

#### 2.3.1. Basic Performance Tests

##### Particle Size Distribution Test

Particle size distribution was measured using a laser particle size analyzer [27] to determine the mass fraction of each particle size range. Samples were reduced using the quartile method, dried at 60 ± 5 °C, and lightly dispersed by gentle pounding. Deionized water was used as the dispersion medium (solid-to-liquid ratio 0.1–0.2%), and ultrasonic dispersion was applied for 60–120 s prior to measurement. Each sample was tested in triplicate, and retesting was performed if the coefficient of variation exceeded 5%. Characteristic particle sizes ($D_{10}$, $D_{30}$, $D_{50}$, $D_{60}$) were determined from the volume distribution and cumulative curves. The uniformity coefficient ($C_{u}$) and curvature coefficient ($C_{c}$) were calculated. The results are presented as curves and parameter tables, with mass balance error ≤ 1%. Calculations were performed according to Equations (1) and (2).

(1) $$C_{u}=D_{60}/D_{10}$$

(2) $$C_{c}=D_{30}^{2}/(D_{10}D_{60})$$

where $D_{10}$, $D_{30}$, and $D_{60}$ represent the particle sizes corresponding to cumulative passing rates of 10%, 30%, and 60%, respectively.

##### Liquid and Plastic Limit Test Method

The liquid and plastic limit tests were conducted in accordance with the relevant standard method for fine-grained soils, using the combined liquid–plastic limit cone penetration approach [28]. After sieving through a 0.5 mm standard sieve, 600 g of the sample was divided into three portions with different moisture contents. The portions were placed in soil containers, covered with damp cloths, and sealed for at least 18 h. Testing was performed using a GYS-2 combined liquid and plastic limit tester. Each sample was placed into a soil cup in layers, compacted, and leveled. After leveling the instrument, the cone tip was gently placed on the soil surface. Penetration depth was recorded 5 s after the start of testing. Each moisture content level was tested in duplicate. Subsequently, a soil sample weighing over 10 g was collected from outside the cone track to determine its moisture content. This procedure was performed on all three specimens to establish a parallel set. A curve was fitted from the w–d data, and the liquid limit ($W_{L}$), plastic limit ($W_{P}$), and plasticity index ($I_{P}$) were calculated based on the cone penetration depth according to the procedure. The results are reported as “mean ± standard deviation.” If the parallel variation exceeds allowable limits, additional tests were conducted, and the reasons were documented. Calculations were performed according to Formula (3).

(3) $$I_{P}=W_{L}-W_{P}$$

##### Determination of Optimum Moisture Content and Maximum Dry Density

The compaction curve for each mixture ratio was determined using the standard Proctor compaction method to establish the optimum moisture content (OMC) and maximum dry density (MDD) [29]. Specimens were pretreated and allowed to settle before testing. Each mixture ratio group included no fewer than five moisture content levels, with at least two parallel tests per level. Compaction was performed in layers using a corresponding circular mold and compaction hammer to ensure uniform density. The surface was leveled to align with the mold rim. Wet density was calculated using Equation (4), and moisture content (w) was determined through simultaneous sampling. Dry density ($\rho_{d}$) was calculated using Equation (5). Compaction curves were plotted from the $\rho_{d}$-w data, with the peak dry density identified as MDD and its corresponding moisture content recorded as OMC.

(4) $$\rho_{wet}=m_{wet}/V$$

(5) $$\rho_{d}=\rho_{wet}/(1+w)$$

where $m_{\mathrm{wet}}$ denotes the wet mass measured after demolding, V represents the mold volume, w indicates the determined moisture content, $\rho_{\mathrm{wet}}$ signifies the wet density, and $\rho_{d}$ denotes the dry density.

##### California Load Ratio Test Method

After preparing the specimen with the initial mix ratio, cover both sides completely with filter paper. Place the specimen base on the porous pressure plate of the fixed rod and secure it firmly. Install four semicircular load plates on top, attach a dial indicator, and record the initial reading. Place the specimen into a water tank and slowly fill until the water level reaches 25 mm above the top of the mold cylinder. Maintain immersion for four days, periodically monitoring the dial gauge reading and assembly stability. After immersion, record the final reading, remove the specimen, allow it to rest for 15 min, and weigh its wet mass [30]. For the penetration test, employ a calibrated force ring and a 50 mm-diameter penetration rod. Apply load using the road strength tester, record force values at 2.5 mm and 5.0 mm penetration depths, convert them to unit pressure, and calculate the CBR following the standard procedure. Take the larger of the two values as the final result. Include parallel specimens in each group and verify both mass and readings. Re-test any abnormal data and document the reasons.

##### Free Expansion Rate Test Method

The free expansion rate (FS) was determined according to JTG 3430—2020. Titanium gypsum and clay were mixed at the specified ratio, crushed, and sieved through a 0.5 mm standard sieve. Approximately 50 g of the sieved sample was placed in an aluminum box, dried at 60 ± 5 °C until a constant weight was reached, and then cooled to room temperature. The sample was transferred into a volumetric flask and leveled, and its mass $m_{0}$ was recorded. The flask volume was calibrated with water to obtain $V_{0}$. The entire sample was transferred through a funnel into a graduated cylinder, to which 30 mL of pure water and 5 mL of NaCl solution were added before pouring in the sample. The mixture was stirred thoroughly with a glass rod, and the liquid level was adjusted to approximately the 50 mL mark using pure water. The cylinder was allowed to settle at 20 ± 2 °C, and the soil column volume $V_{t}$ was recorded every 2 h. When the difference between two consecutive readings was ≤0.2 mL, the stable volume $V_{eq}$ was recorded. At least three parallel tests were performed for each mix ratio. The results are reported as “mean ± standard deviation,” documenting $m_{0}$, $V_{0}$, NaCl solution details, ambient temperature, and reading intervals. The free expansion rate was calculated using Formula (6) and expressed as a percentage.

(6) $$FS=\frac{V_{eq}-V_{0}}{V_{0}}\times 100\%$$

where $V_{eq}$ represents the volume after stabilization, and $V_{0}$ denotes the volume of the soil measuring cup calibrated with water.

##### Test Method for Water Stability Coefficient

Cylindrical specimens (Φ 38 mm × 80 mm) were prepared using the static compaction method according to JTG 3441—2024, ensuring a height-to-diameter ratio greater than 2, and subsequently subjected to standard curing. After reaching the designated curing age (T-1), specimens were immersed in deionized water. The unconfined compressive strength (UCS) of specimens TSC1–TSC5 was measured using a fully automatic UCS testing machine [31]. Water stability was assessed via the water stability coefficient K, calculated according to Equation (7).

(7) $$K=S_{w}/S$$

where $S_{w}$ denotes the unconfined compressive strength (UCS) after 1 day of immersion at a curing age of T-1, and S denotes the UCS at a curing age of T.

#### 2.3.2. Micro-Testing Methods

##### Product Phase Analysis Testing Methods

An X-ray diffractometer (TD-3500) [32] was used to analyze the crystalline phases of TS, TSC, and TSC-J. Samples were irradiated with a Cu-Kα target over a 5–85° scanning range at 0.3° per second. To further identify functional groups in the materials, a Fourier Transform Infrared Spectrometer (FTIR-650S) [33] was used to complement the XRD analysis of metallic and non-metallic crystalline phases. KBr pellets were prepared for measurement, and scanning was divided into background and sample acquisition phases, totaling 64 steps. The instrument resolution and lens rate were set to 2.0 cm^−1^ and 0.625, respectively.

##### Test Method for Product Quantity Relationship

The carbon fixation capacity of C-PHCP was determined using a HTG-2 thermogravimetric analyzer [34]. Approximately 10 mg of sample was weighed into an alumina crucible and heated from room temperature to approximately 1000 °C at a heating rate of 10 °C/min. Weight loss at different temperatures was recorded using a precision electronic balance.

##### Testing Methods for Microstructural Characteristics

The microstructures of TS, TSC, and TSC-J were examined using a COXEM EM-30 scanning electron microscope (SEM). The elemental composition was analyzed using a QUANTAX XFlash energy dispersive spectrometer (EDS) [35]. Owing to the poor electrical conductivity of the samples, they were uniformly coated with gold via ion sputtering before testing. After mounting the samples on the stage, a high vacuum was established. Imaging was conducted by selecting suitable acceleration voltages and working distances. EDS point or area spectra were collected simultaneously when required. The specific surface area and pore structure were measured using the nitrogen adsorption–desorption method (BET) [36]. To prevent structural alterations in gypsum hydrates and MICP products, powder samples underwent vacuum degassing to remove physically adsorbed water and volatile substances. Measurements were carried out at 77 K (liquid nitrogen), and adsorption–desorption isotherms were recorded at P/P_0_ = 0.01–0.99. These data were used for comparison and cross-validation with SEM/EDS morphological observations.

## 3. Results and Discussion

### 3.1. Basic Properties of Clay-Modified Titanium Gypsum

#### 3.1.1. Particle Size Distribution

**Figure 2:**
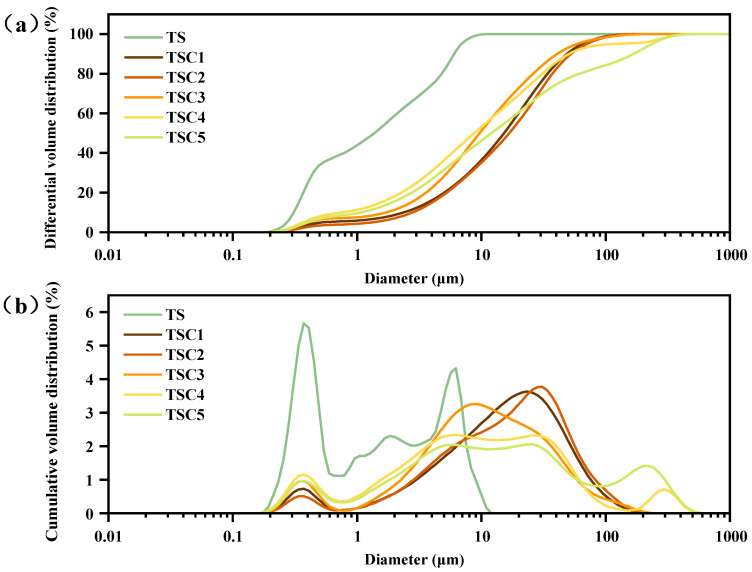
Particle Gradation at Different Clay Content Levels: (**a**) Differential Volume Distribution Map; (**b**) Cumulative Volume Distribution Plot.

Particle gradation is a key indicator describing the distribution of particles across different size ranges. It directly affects the bulk density, pore structure, and particle contact state of the system, thereby strongly influencing the material’s physical and mechanical properties and its engineering applicability. Preliminary tests indicated that the original titanium gypsum particles displayed a dispersed size distribution with an uneven mixture of coarse and fine particles. This distribution resulted in a loose skeletal structure and high pore connectivity, which limited the material’s fundamental engineering performance. To mitigate this issue, clay was incorporated to adjust the particle size distribution of titanium gypsum. Figure 2 presents the particle size distribution at varying clay dosages.

As clay content increases, the particle frequency curve shifts leftward and broadens, accompanied by a notable increase in the volume fraction of fine particles and a simultaneous decrease in characteristic particle sizes D_10_, D_30_, D_50_, and D_60_. The slope of the cumulative distribution curve on a logarithmic scale gradually flattens, reflecting the progressive filling of medium and coarse particles by fine materials [37]. At clay contents of 10–30%, the original “bimodal” particle size distribution gradually shifts to a “broad-based unimodal or weakly bimodal” pattern, indicating preliminary compatibility between skeletal and interstitial particles. At around 40% clay content, the particle size distribution curve exhibits a full-peak shape with moderate width, and the cumulative distribution curve remains continuous and smooth, indicating optimal aggregate grading and maximum potential bulk density. Further increasing the clay content to 50% leads to a sharp, narrow single-peak distribution curve, suggesting narrowed grading and insufficient skeletal particles [38]. This indicates that the uniformity coefficient and curvature coefficient follow a “first increase, then decrease” trend with changing clay content, reaching optimal values near 40%. This geometric restructuring not only increases interparticle contacts and reduces pore neck dimensions but also enhances pore continuity, providing a structural basis for the subsequent optimization of water retention and improvement of mechanical properties [39].

#### 3.1.2. Liquid Limit and Plastic Limit

**Figure 3:**
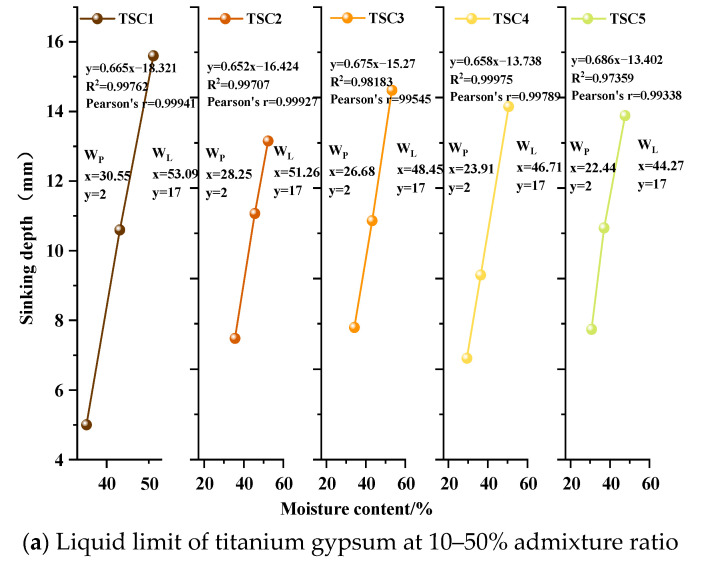

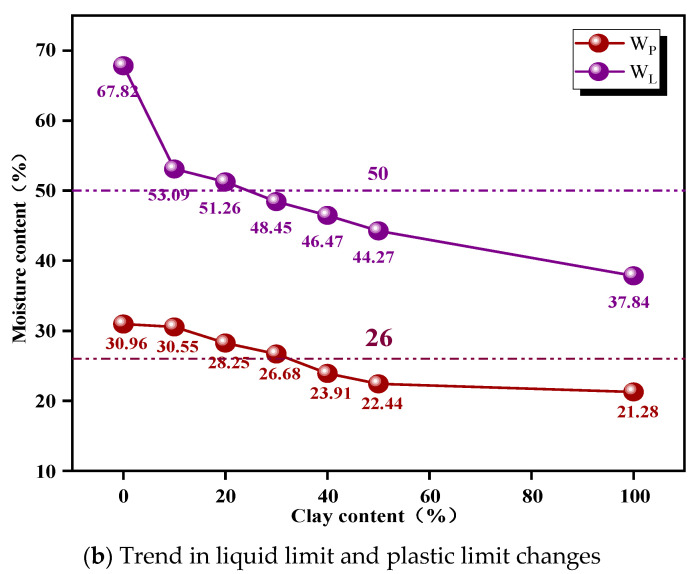
Summary of Liquid and Plastic Limits of Titanium Gypsum at Different Clay Content Levels.

The liquid limit (W_L_) and plastic limit (W_P_) are primary indicators describing the plasticity of fine-grained materials. The liquid limit represents the critical moisture content at which plasticity transitions to a fluid state, whereas the plastic limit denotes the critical moisture content at which a semi-solid state transitions to a plastic state. Their values directly affect the mechanical properties of the material [40]. Preliminary experiments indicated that titanium gypsum has uneven particle grading and a high liquid limit, resulting in substantial volume deformation upon water absorption, thereby limiting molding quality and engineering applicability. This study systematically examined the variations in liquid and plastic limits at different clay addition levels to optimize the titanium gypsum system. The results (Figure 3b) indicate that, with increasing clay content, the liquid limit decreases from 67.82% to 37.84% and the plastic limit decreases from 30.96% to 21.28%, both showing a consistent downward trend. At clay contents of 20–30%, the W_L_ falls below the 50% threshold for high plasticity, indicating a transition of the system from high to low plasticity. At 30–40% clay content, the W_P_ decreases below 26%, narrowing the plastic range and enhancing strength at the critical moisture content. The plasticity index I_P_ (W_L_−W_P_) decreases from 36.86% to approximately 22% at clay contents of 10–50%, and further to 16.6% at 100% content, indicating controlled overall plasticity and reduced water absorption sensitivity [41]. The “de-plasticizing” effect primarily arises from two mechanisms. First, clay, as a low-to-medium plasticity fine material, dilutes highly plastic titanium gypsum, reduces free water adsorption, and compensates for deficiencies in coarse-grained particle gradation. This process forms a uniform skeleton, thereby enhancing particle contact stiffness and overall system stability [42]. In summary, clay admixture markedly reduces the liquid and plastic limits through physical gradation optimization, providing a structural basis for subsequent enhancements in mechanical properties and water retention performance.

#### 3.1.3. Optimum Moisture Content and Maximum Dry Density

**Figure 4:**
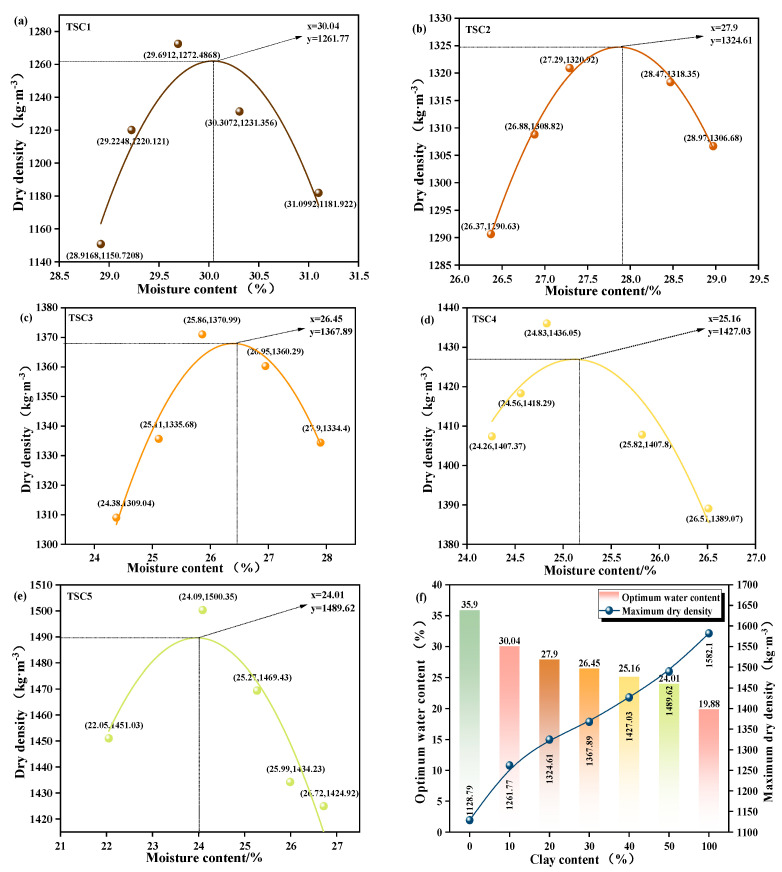
OMC and MDD Values of Titanium Gypsum at Different Clay Content Levels: (**a**–**e**) OMC and MDD Values at 10~50% Clay Content; (**f**) Summary Comparison Chart.

The optimum moisture content (OMC) and maximum dry density (MDD) are primary indicators for assessing the compaction performance of powdered materials. They directly affect construction compaction efficiency, compacted density, and subsequent mechanical stability, making them critical parameters that require strict control in engineering applications of titanium gypsum. Previous studies have demonstrated that clay content regulates the liquid and plastic limits of titanium gypsum. Variations in these limits inevitably affect the material’s moisture requirements and compaction potential. Based on this, the present study further examined the evolution of OMC and MDD in titanium gypsum at varying clay contents, with results presented in Figure 4a–e. As clay content increases, both OMC and MDD exhibit typical bell-shaped trends, with peak parameters determined using quadratic polynomial fitting [43]. When clay content increased from 0% to 50%, OMC decreased monotonically, whereas MDD increased approximately monotonically, indicating a reduced moisture requirement and enhanced compaction potential. Notably, at 30% clay content, the maximum dry density reached 86.90% of that of clay [44], reflecting optimized particle grading and structure, which enhances compactability and achieves high density. Further analysis of Figure 4f reveals that OMC decreases nearly linearly with increasing clay content, dropping from 35.9% to 24.01%—a reduction of approximately 11.9 percentage points—while MDD increases nearly linearly from 1213.8 kg·m^−3^ to 1489.6 kg·m^−3^, an increase of approximately 22.7%. This opposite trend aligns with the decreasing liquid and plastic limits, indicating that clay reduces the material’s moisture requirement through fine-particle filling, while simultaneously enhancing compacted density and strength.

#### 3.1.4. California Load Ratio

**Figure 5:**
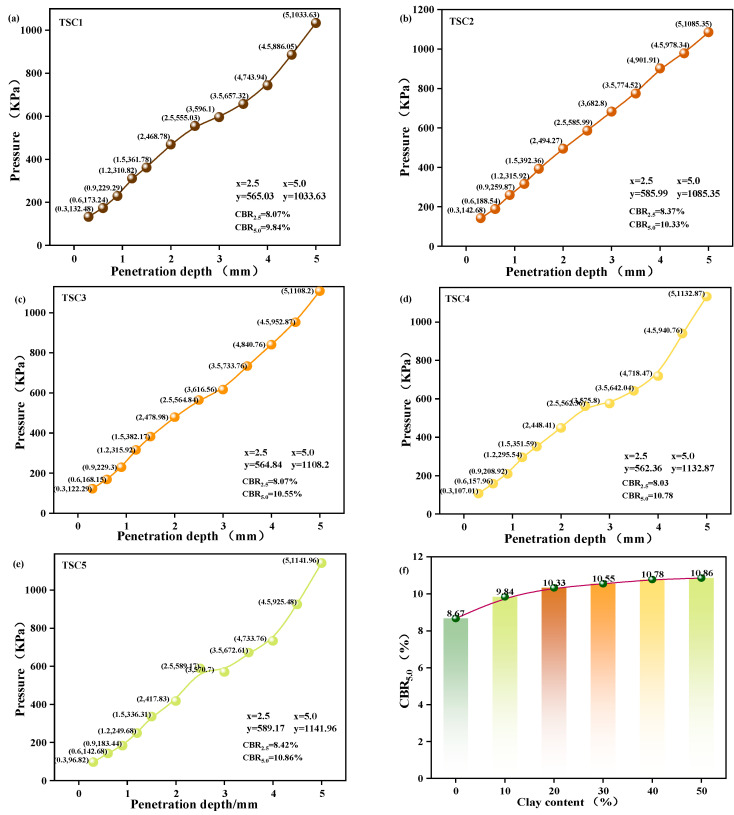
CBR Values of Titanium Gypsum at Different Clay Content Levels: (**a**–**e**) CBR Values at 10~50% Clay Content; (**f**) Summary Comparison Chart of CBR Values.

The California Bearing Ratio (CBR) is a primary indicator for assessing the bearing capacity and deformation resistance of subgrade and foundation materials. Its value directly reflects a material’s ability to resist penetration deformation under load, serving as a critical criterion for determining whether titanium gypsum satisfies engineering bearing requirements [45]. Previous studies have demonstrated that clay content enhances material properties by optimizing particle gradation, regulating liquid and plastic limits, and improving compaction characteristics, including OMC and MDD. These improvements further affect the material’s bearing capacity. Therefore, this study systematically investigated the variation in titanium gypsum bearing capacity at different clay contents using CBR testing [14], with results presented in Figure 5. As clay content increases, CBR values exhibit an upward trend, progressively increasing with penetration depth. This trend reflects enhanced inter-particle contact forces and improved material bearing capacity. Specifically, when clay content increases from 10% to 50%, CBR values increase significantly [46]. Within the 20–40% clay content range, the curve exhibits the steepest slope, indicating the most pronounced improvements in compaction efficiency and bearing capacity. Beyond 40% clay content, CBR values stabilize, suggesting that material strength reaches saturation, and further clay addition has a limited effect on bearing capacity enhancement. From the perspective of particle gradation, increasing clay content progressively optimizes the internal structure, enhances particle contact density, and improves compaction efficiency [47]. The optimal clay content range is 30–40%, where gradation is most appropriate, achieving optimal compaction and structural stability, accompanied by a significant increase in CBR values. Excessive clay content, however, results in insufficient skeletal particles, narrower gradation, and saturated compaction efficiency, limiting further performance improvement from additional clay. In summary, the optimal clay content range for titanium gypsum is 30–40%, where the material achieves an optimal balance between bearing capacity and compactability.

#### 3.1.5. Free Expansion Rate

**Figure 6:**
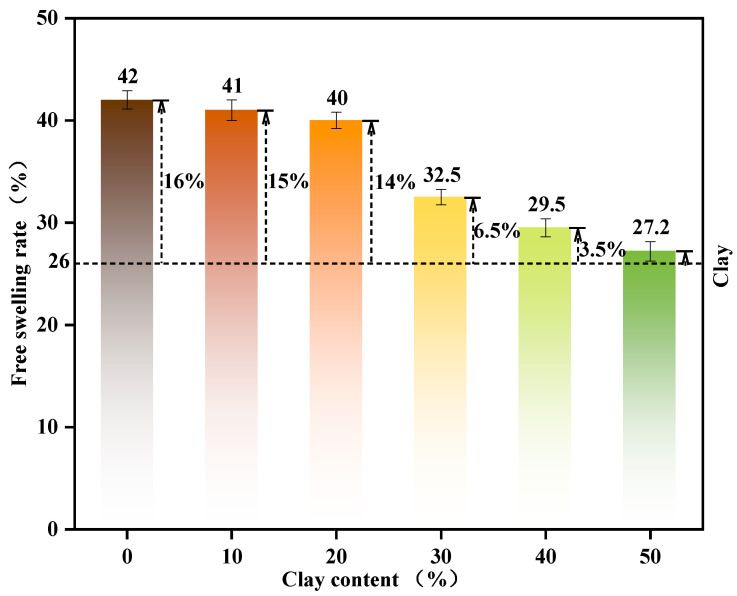
Summary of Free Swelling Rates for Titanium Gypsum Blends with Clay.

The free expansion rate is a primary indicator reflecting the volume stability of fine-grained soils and industrial solid waste materials, assessing their expansion deformation under unconfined water absorption conditions. This property directly affects the structural integrity and load-bearing safety of engineering works, making it a critical consideration in titanium gypsum engineering applications. Previous studies have confirmed that increasing clay content optimizes the particle size distribution of titanium gypsum and enhances its bearing capacity. However, the response of volume stability to varying clay content remains poorly understood. Therefore, this study investigates the regulatory effect of clay content on the free expansion rate of titanium gypsum to elucidate the mechanism underlying its enhanced volume stability. Figure 6 shows that the free expansion rate of titanium gypsum decreases markedly with increasing clay content, from 41% at 10% clay content to 27.2% at 50% clay content. This reduction is attributed to clay filling particle voids, enhancing particle bonding strength, reducing water infiltration and available expansion space, thereby suppressing volumetric expansion. Pure titanium gypsum, owing to its high expansivity, is unsuitable for engineering applications demanding high volume stability. Incorporating clay markedly reduces expansivity, with the most favorable effect observed at clay contents of 30–50% [48]. In summary, adjusting clay content effectively regulates the expansion behavior of titanium gypsum-based materials, facilitating targeted optimization of engineering properties. This principle provides theoretical support for titanium gypsum engineering applications and serves as technical guidance for subsequent mix design optimization and experimental planning. It should be noted that the laboratory tests were conducted under controlled conditions, and some factors present in real engineering environments were not considered, such as cyclic wetting–drying processes, long-term environmental exposure, stress conditions, and chemical interactions with surrounding soils or groundwater. These factors may influence the expansion behavior and long-term stability of the material in field applications.

**Figure 7:**
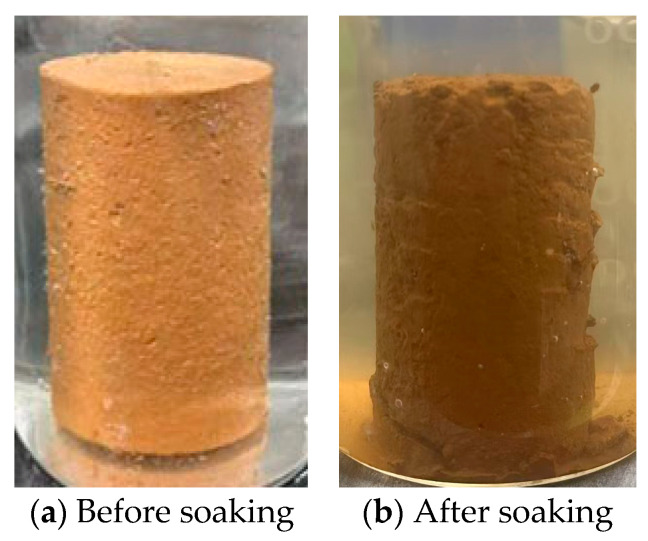
Morphological Comparison of Titanium Gypsum Before and After Water Immersion.

Native titanium gypsum exhibits substantial performance deficiencies, including imbalanced particle size distribution, elevated liquid and plastic limits, high expansion rates, and insufficient bearing capacity, rendering it unsuitable for meeting the stability and mechanical requirements of highway subgrade fill materials. To enhance its applicability, this study employs clay blending modification, systematically investigating the regulatory effects of clay dosage on five core indicators to elucidate performance evolution patterns and identify the optimal dosage. As clay content increases, particle gradation progressively improves, providing a structural foundation for enhanced mechanical properties. Liquid and plastic limits exhibit a pronounced “de-plasticization” trend: both limits decrease with increasing clay content, while the plasticity index diminishes and stabilizes at moderate clay levels. Compaction properties improve synchronously: optimum moisture content and maximum dry density display a bell-shaped distribution. Within the primary addition range, optimum moisture content decreases linearly, whereas maximum dry density increases significantly. At medium addition levels, maximum dry density approaches that of clay, confirming the critical role of fine-grain filling in enhancing compactability. Concurrently, CBR values initially increase rapidly with increasing clay content before stabilizing, with the most pronounced gains observed at low-to-medium and medium clay content levels. At these points, optimal gradation achieves maximum compaction and skeletal stability. However, at high clay content, gains diminish due to insufficient skeletal support, defining an optimal clay content that balances compaction and load-bearing capacity. Regarding volume stability, the free expansion rate continuously decreases with increasing clay content, with the most pronounced suppression observed at medium-to-high dosages. Clay effectively mitigates the high expansion defect inherent in native titanium gypsum by filling pores, enhancing particle bonding, and reducing water infiltration, thereby enabling controlled volume deformation. In summary, clay dosage significantly modulates all five properties, with medium dosages providing the most comprehensive optimization. Considering particle gradation, plasticity, compaction bearing capacity, and volume stability, the TSC with 40% clay content is selected as the optimal composite system. However, preliminary experiments revealed that its structure loosened, strength sharply declined, and it even disintegrated after water immersion (Figure 7), indicating that the water-solubility defect remained unresolved. To address this issue, microbial communities were introduced to induce carbonate precipitation for pore filling, thereby enhancing the performance of the composite material [49].

### 3.2. Research on Water Stability Based on Microbial Mineralization

#### 3.2.1. Mechanism of Calcium Source Influence on System Water Stability Evolution

**Figure 8:**
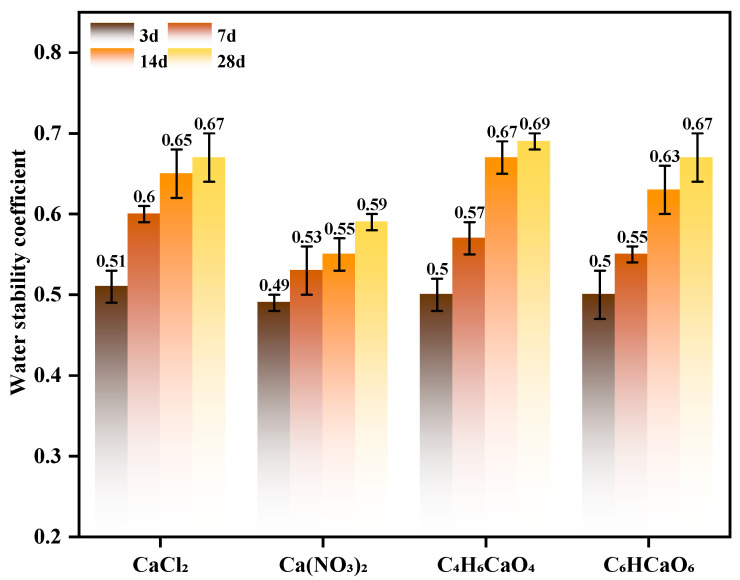
Effect of Calcium Source Category on Water Stability.

To investigate the effect of calcium source type on the water stability of TSC composites, this study selected four representative calcium sources—calcium chloride, calcium nitrate, calcium acetate, and calcium lactate—for comparative experiments [50]. The water stability coefficient was employed as the key evaluation metric to quantify the effect of different calcium sources on the material’s resistance to water immersion. The results are presented in Figure 8. The experiments demonstrated that calcium chloride markedly enhances the material’s water stability. Although initial stability was low, water stability progressively improved with extended curing time, showing pronounced long-term enhancement after 28 days. This phenomenon is primarily attributed to the high solubility and reactivity of calcium chloride in water. Upon calcium ion release, a dense crystalline structure forms between particles, thereby enhancing the material’s internal cohesion [51]. In contrast, calcium nitrate exhibited limited improvement in water stability at both 14 and 28 days. Its rapid dissolution may lead to incomplete reactions and unstable bonding with other mineral phases, thus limiting long-term water stability improvement. Calcium acetate [52] and calcium lactate displayed relatively gradual changes in water stability. In particular, calcium lactate, owing to its low solubility and slow calcium ion release, was unable to significantly enhance material water stability. Furthermore, the resulting precipitate structure may be less dense than that formed by calcium chloride, thus limiting its effectiveness in improving volume stability [51]. From a cost-effectiveness perspective, calcium chloride provides a simpler production process, abundant raw materials, and large-scale supply capacity, rendering it considerably more economical than calcium nitrate, calcium acetate, or calcium lactate. The latter compounds involve complex production and storage requirements, high procurement and handling costs, and require meticulous management during use to prevent adverse reactions, leading to overall higher costs. Calcium lactate, due to its complex preparation process and low reactivity, produces negligible effects, further limiting its applicability. In summary, calcium chloride combines superior water stability enhancement with cost advantages, making it the preferred calcium source for optimization in this study. It was subsequently employed as the sole calcium source in further experiments to validate its suitability under various application conditions [53].

#### 3.2.2. Mechanism of Temperature Influence on the Evolution of System Water Stability

**Figure 9:**
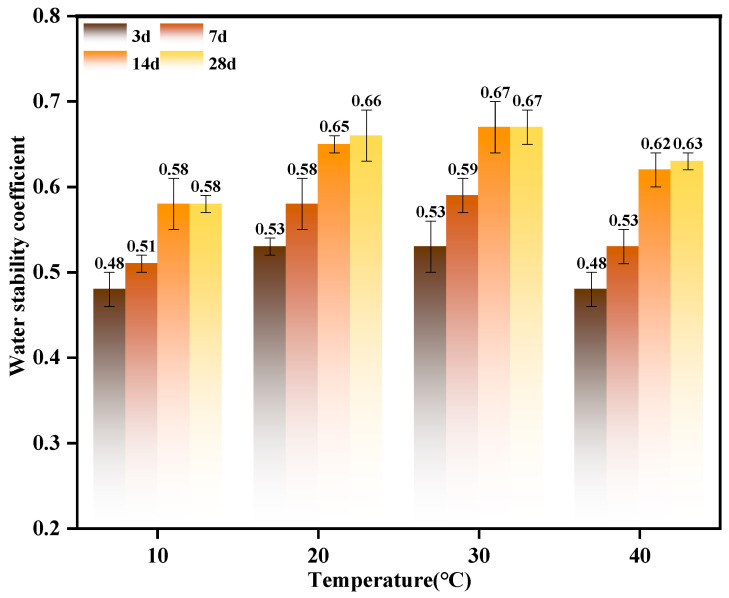
Effect of Temperature on Water Stability.

To elucidate the regulatory effects of temperature on material water stability and determine the optimal temperature range, this study conducted systematic water stability tests under varying temperature conditions (Figure 9). The results indicate that temperature markedly influences water stability, showing an initial increase followed by a subsequent decrease. At low temperatures (10 °C and 20 °C), the material exhibited low water stability coefficients, approximately 0.48 and 0.51 at 3 and 7 days, respectively, indicating that low temperatures hinder the development of water stability. This effect is attributed to low temperatures inhibiting chemical reaction rates and mineral hydration, thereby reducing effective particle contact and bonding forces and weakening structural stability [25]. At 30 °C, water stability reaches its peak, with test values at 14 and 28 days approximately 0.65 and 0.66, respectively, indicating that moderate temperatures enhance water stability. This temperature accelerates hydration and calcium carbonate precipitation, while balancing crystal density and system stability, enabling MICP or mineralization-modified systems to achieve optimal stability [54]. At 40 °C, water stability slightly declines, reaching approximately 0.62 at 28 days. Excessively high temperatures exert adverse effects. Potential mechanisms include accelerated water evaporation, thermal rearrangement or disruption of precipitated crystal structures, and increased stress at crystal–particle interfaces, leading to microcracks—all of which are detrimental to long-term stability. In summary, the regulation of water stability by temperature in this system follows an initial increase followed by a decrease, with 30 °C being the optimal temperature to maximize structural stability in aquatic environments [55]. This demonstrates that temperature control is a critical operational parameter in applying MICP or mineralization-modified materials, necessitating optimization in engineering practice.

#### 3.2.3. Mechanism of Ph Influence on the Evolution of System Water Stability

**Figure 10:**
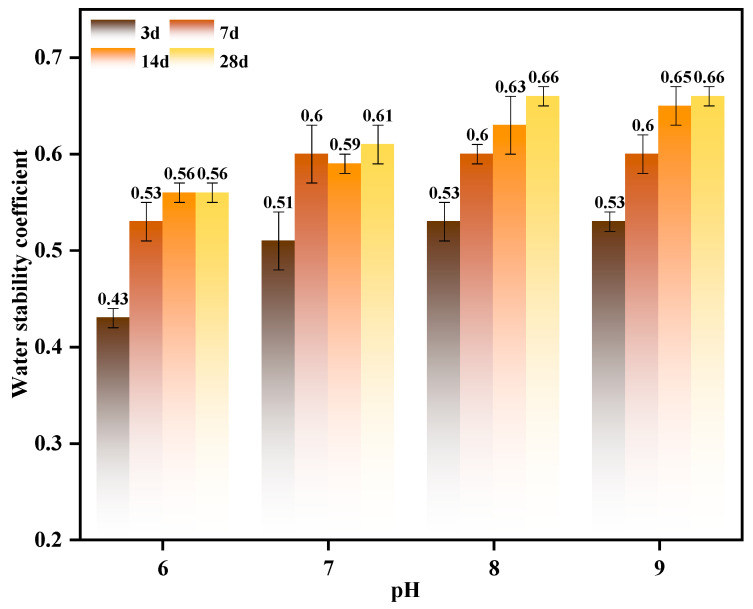
Effect of pH on Water Stability.

To elucidate the regulatory mechanism of pH on the water stability of the material and identify the optimal range for performance enhancement, systematic water stability tests were conducted under varying pH conditions. As shown in Figure 10, low pH conditions markedly impaired the material’s water stability, with coefficients of approximately 0.43 and 0.51 at 3 and 7 days, respectively, indicating that acidic environments substantially compromise structural integrity. This effect primarily results from low pH increasing mineral particle solubility, weakening interparticle contact forces, and inhibiting the formation of hydration products and cementation, thereby reducing structural density and water erosion resistance [56]. Water stability progressively improves as pH increases. This improvement is particularly pronounced at pH 8, where water stability coefficients at 14 and 28 days increase to approximately 0.59 and 0.60, respectively. This indicates that a neutral-to-alkaline environment provides optimal conditions for cementation reactions. At this pH, calcium ions can fully react with other ions in the solution to form relatively stable hydrates and cementation products, promoting effective interparticle bonding and enhancing overall structural density. Even at pH 9, water stability remained high, with coefficients further increasing to approximately 0.63 and 0.66 at 14 and 28 days, respectively. The elevated alkalinity continuously promotes calcium source precipitation and cementation phase formation, thereby constructing a more robust mineral structure and enhancing the material’s resistance to water damage and long-term durability [57]. In summary, pH markedly influences the material’s water stability. Acidic conditions substantially weaken stability, whereas neutral to alkaline pH ranges significantly enhance the formation and persistence of stable structures. Therefore, adjusting the system pH to an appropriate range is crucial for achieving high water stability and long-term service performance in material regulation and engineering applications.

#### 3.2.4. Mechanism of Admixture Content on the Evolution of System Water Stability

**Figure 11:**
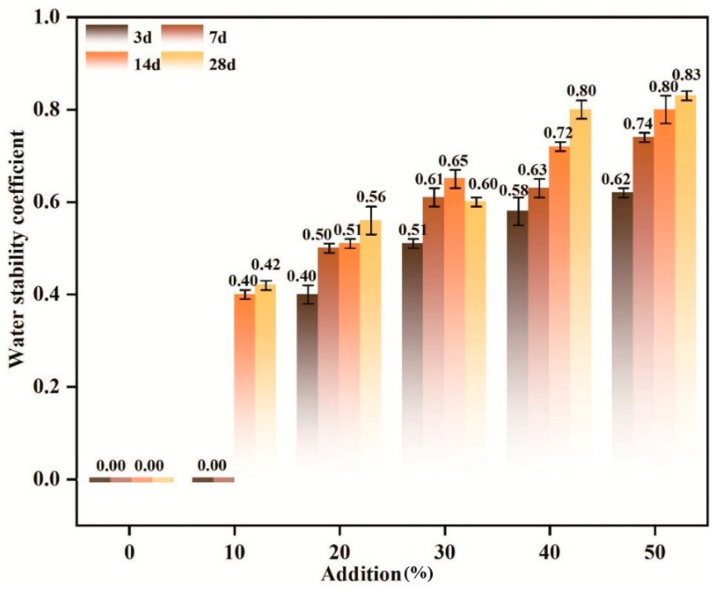
Effect of Microbial Suspension Dosage on Water Stability.

To elucidate the regulatory effects of bacterial suspension dosage on material water stability and identify the optimal range balancing modification efficiency and microbial activity, this study maintains calcium chloride as the calcium source, controlled system pH at 9, and kept the temperature at 30 °C in single-factor experiments, adjusting only the bacterial suspension dosage to eliminate interference from other environmental variables. Figure 11 shows that bacterial suspension dosage significantly influences water stability, exhibiting strong time dependency and dosage sensitivity. During the early stages (3 and 7 days), the low-dosage group (0–10%) exhibited relatively low water stability, indicating that the microbial community had not yet developed structurally complete or functionally mature biofilms on particle surfaces. Insufficient extracellular polymer secretion (EPS) and weak adhesion [58] limited localized Ca^2+^/CO_3_^2−^ enrichment, restricting carbonate nucleation and early precipitation, thereby inhibiting water stability development [59]. As the bacterial suspension dosage increased, microbial density rose, biofilm formation accelerated, quorum sensing was activated, and enhanced metabolic activity promoted more sustained and efficient mineralization reactions. Within the moderate inoculum range (approximately 30–50%), water stability significantly improved at both 14 and 28 days [60], indicating that appropriate inoculum levels promote effective calcium carbonate formation and intergranular cementation, thereby markedly enhancing structural integrity [26]. However, further increases in bacterial suspension dosage resulted in only marginal improvements in water stability. This is primarily due to high cell density, which causes precipitation localization imbalances. Excessively thick biofilms, precipitation at non-critical stress interfaces, premature pore blockage, and impaired mass transfer of nutrients and reactants [61] ultimately undermine sustained mineralization capacity. Moreover, accelerated metabolic processes may lead to NH_4_^+^ accumulation and localized pH fluctuations, potentially inhibiting stable calcium carbonate crystal formation and partially dissolving early precipitates, thereby negatively impacting long-term water stability [62]. In summary, bacterial loading exhibits a “first-boost-then-flattening” pattern in regulating water stability. At low dosages, limitations arise from insufficient biofilm development and mineralization, whereas moderate dosages achieve peak mineralization efficiency and optimal cementitious structure. Excessive dosages reduce water stability gains due to mass transfer limitations and metabolic side effects. Therefore, engineering applications should determine optimal microbial suspension dosages based on microbial activity and the spatial distribution of mineralization products as key metrics. These dosages should be synergistically optimized with environmental factors such as temperature and pH to ensure sustained enhancement of material water stability and long-term durability.

### 3.3. Analysis of Microbial Mineralization Mechanisms

#### 3.3.1. Product Phase Analysis

**Figure 12:**
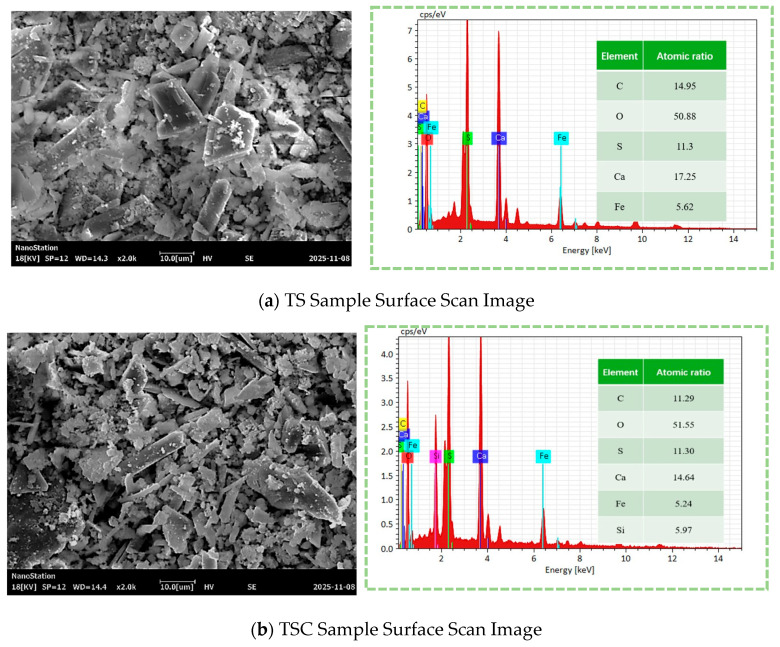

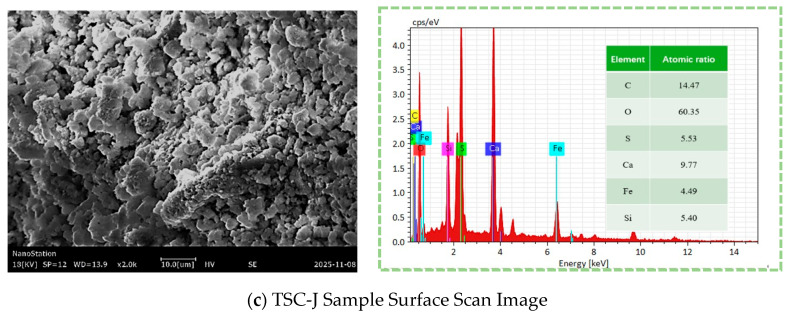
Comparison of TS, TSC, and TSC-J Samples at Different Magnifications.

To elucidate the regulatory mechanism of microbial mineralization on the phase composition of the TSC system and to clarify the phase evolution and formation characteristics of newly formed minerals during solidification, this study systematically characterized native titanium gypsum (TS), composite materials with 40% clay (TSC), and materials after microbial solidification (TSC-J) using SEM, FTIR, and XRD. Figure 12 presents SEM and EDS results for the three sample groups, visually illustrating significant changes in surface morphology and elemental composition across different treatments. The TS surface exhibits regular plate-like and columnar crystal structures with well-developed intergranular pores and a relatively loose framework, consistent with the typical plate-like or needle-like crystals of CaSO_4_·2H_2_O [63]. EDS analysis indicates that Ca and S are the primary elements, accounting for 23.9 wt.% and 11.3 wt.%, respectively, while minor Fe signals originate from iron oxide impurities in the raw material. No significant Si or Al signals were detected, confirming the absence of clay introduction or notable phase reactions at this stage. After incorporating 40% clay, the TSC sample shows a markedly altered microstructure. Portions of the original titanite crystals were partially encapsulated or filled, with particles arranged more densely. Layered stacking structures were observed in localized areas, indicating the formation of interfacial bonding within the composite. EDS analysis revealed substantial increases in Si and Al content (approximately 7.0 wt.% and 3.7 wt.%, respectively), indicating that SiO_2_ and Al_2_O_3_ from the clay provided skeletal support and structural regulation within the composite system. Concurrently, the proportions of Ca and S decreased, reflecting a relative dilution of the gypsum components. This composite structure improved particle and pore distributions, providing favorable nucleation and deposition sites for subsequent microbial-induced mineralization. After Bacillus consolidation, the TSC-J surface further densified, with original pores extensively filled by deposits forming a continuous cementation layer. Irregular agglomerate-covered structures appeared in localized areas, interpreted as microbially induced calcium carbonate precipitation [64]. Compared to TSC, EDS analysis revealed Ca content increased to approximately 24 wt.%, C content rose significantly to about 15 wt.%, and S content decreased to 5.5 wt.%. This shift indicates that a portion of Ca^2+^ in the system reacts with CO_3_^2−^ through microbially induced mineralization, forming stable CaCO_3_ deposits that achieve secondary cementation and pore filling of titanite particles. This mineralization appears to enhance structural density and integrity, thereby providing a possible basis for improving the material’s macroscopic mechanical properties and water stability.

**Figure 13:**
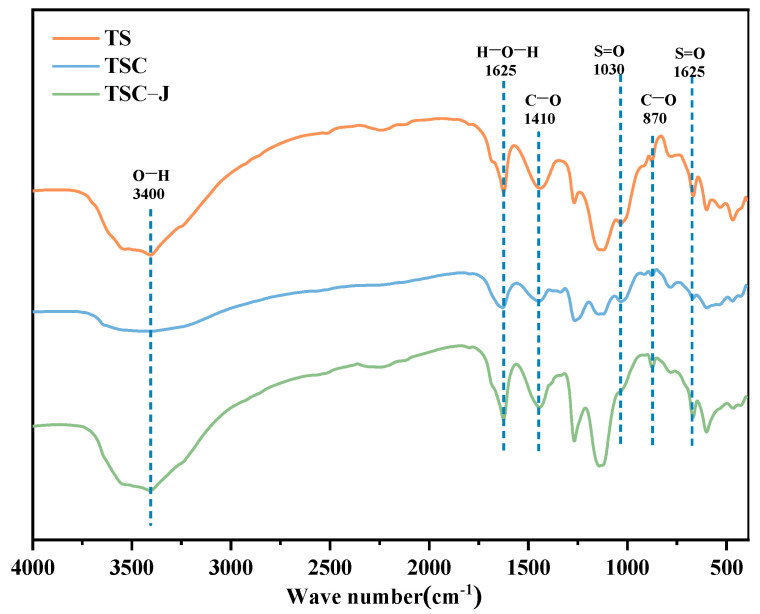
Infrared Spectra of TS, TSC, and TSC-J Samples.

Figure 13 presents the FTIR spectra of TS, TSC, and TSC-J samples. All three samples exhibit major absorption peaks at approximately 3400, 1625, 1410, 1030, and 870 cm^−1^. Variations in peak positions and intensities reflect changes in phase composition and structural characteristics induced by different material treatments [65].

The prominent absorption peak at approximately 3400 cm^−1^ corresponds to –OH stretching vibrations, mainly from crystalline water and adsorbed water in CaSO_4_·2H_2_O. This peak is strongest in TS and progressively weakens in TSC and TSC-J, indicating reduced bound water content due to clay incorporation and partial water immobilization by newly formed minerals after microbial solidification. The peak near 1625 cm^−1^ represents H–O–H bending vibrations, potentially overlapping with S=O stretching vibrations of sulfate ions (SO_4_^2−^). Its progressive weakening indicates reduced moisture content and alterations in the sulfate chemical environment [66].

Distinct C–O stretching and bending peaks at 1410 cm^−1^ and 870 cm^−1^ are characteristic of the CO_3_^2−^ group in CaCO_3_. These peaks are weak in TS and TSC but significantly enhanced in TSC-J, indicating that Bacillus-mediated mineralization promoted substantial CaCO_3_ formation. This result aligns with the mechanism in which microorganisms catalyze urea decomposition via urease to produce CO_3_^2−^, which combines with Ca^2+^ to form CaCO_3_ crystals, confirming biomineralization’s dominant role in structural evolution. Additionally, S=O peaks at 1030 cm^−1^ and 1625 cm^−1^ are markedly weakened and slightly shifted in TSC-J, indicating altered SO_4_^2−^ environments. These changes likely result from ion exchange or signal masking caused by newly formed CaCO_3_ and clay phases [67], suggesting partial structural transformation of the original CaSO_4_ in the system. In summary, FTIR analysis indicates that TS predominantly contains hydrated CaSO_4_; after clay incorporation (TSC), the hydroxyl peak weakens and broadens, reflecting clay-mediated interfacial regulation of the calcium sulfate crystal structure; and, after microbial consolidation (TSC-J), the C–O peak intensifies while the S=O peak weakens, indicating substantial CaCO_3_ formation and partial CaSO_4_ transformation. The material evolves from a single sulfate phase to a carbonate–sulfate composite mineral phase. This phase transformation provides robust microscopic evidence supporting subsequent mechanical strengthening and durability enhancement.

**Figure 14:**
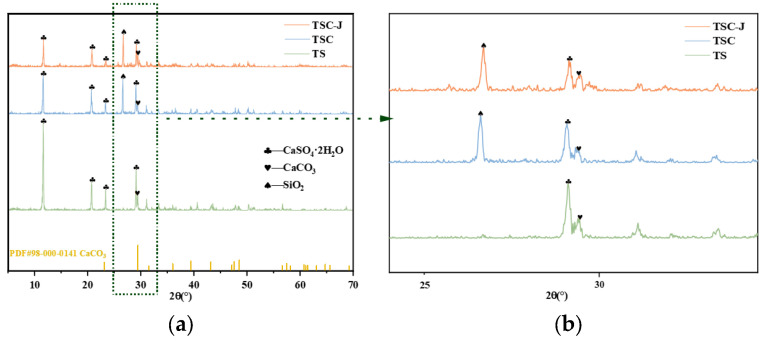
X-ray diffraction patterns of TS, TSC, and TSC-J samples: (**a**) overall XRD patterns; (**b**) magnified view of the diffraction peaks around 2θ ≈ 29°.

Figure 14 shows the XRD patterns of TS, TSC, and TSC-J samples, with Figure 14b representing a local magnification of Figure 14a at 2θ ≈ 29°. Analysis indicates that the main diffraction peaks correspond to three mineral phases: dihydrate gypsum (CaSO_4_·2H_2_O), calcium carbonate (CaCO_3_), and silicon dioxide (SiO_2_). TS exhibits sharp and intense diffraction peaks, with main peaks at 2θ ≈ 11.6°, 20.7°, 23.4°, 29.1°, and 31.1°, indicating it predominantly consists of gypsum dihydrate with high crystallinity and minimal impurities. In TSC, the intensity of gypsum peaks decreases significantly after incorporating 40% clay. Simultaneously, a SiO_2_ peak appears at 2θ ≈ 26.6°, indicating that quartz from clay introduces a new crystalline phase, complicating the system’s mineral composition [68]. In contrast, TSC-J exhibits distinct calcite peaks alongside gypsum and quartz peaks. Notably, the main calcite peak at 2θ ≈ 29.4° overlaps with the gypsum peak at 29.1° (Figure 14b), and secondary calcite peaks are observed in the 39–48° range [69]. This indicates that CaCO_3_ crystals form in the system after Bacillus-mediated curing [70]. Based on the MICP mechanism, urease secreted by bacteria catalyzes urea hydrolysis. The resulting CO_3_^2−^ reacts with Ca^2+^ to form CaCO_3_, leading to the emergence and strengthening of calcite peaks. Intensity changes show that the relative intensity of the CaCO_3_ peak increases in the order TS < TSC < TSC-J, indicating CaCO_3_ content gradually increases with MICP treatment. Concurrently, the intensity of the CaSO_4_·2H_2_O peak in TSC-J decreases, potentially due to partial gypsum dissolution or coverage by CaCO_3_ deposits. No distinct peaks for hemihydrate gypsum or anhydrite are observed, indicating a stable hydration state. Overall, calcite formation in TSC-J suggests the occurrence of MICP-induced solidification in the TSC system. The newly formed CaCO_3_ fills pores and forms bridging structures between particles, enhancing mechanical strength and structural density. This aligns with the significantly enhanced water stability observed in TSC-J.

#### 3.3.2. Product Quantity Relationships

**Figure 15:**
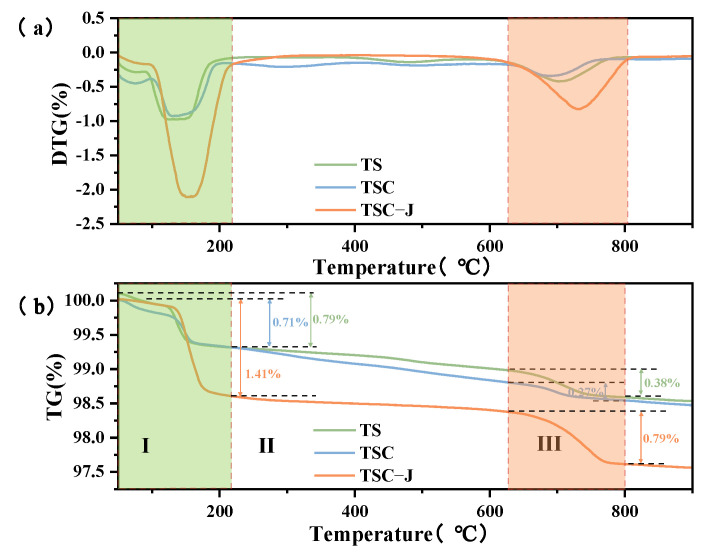
Thermogravimetric Analysis of TS, TSC, and TSC-J Samples (**a**) Differential Thermogravimetric Analysis Curves (**b**) Thermogravimetric Analysis Curves.

To quantify the effect of microbial mineralization on the thermal behavior and product formation of the TSC system, and to clarify the relationship between thermal decomposition of each component and CaCO_3_ content, thermogravimetric analysis (TGA) was performed on TS, TSC, and TSC-J samples. The corresponding TG and DTG curves are shown in Figure 15a,b. Three samples exhibit three-stage thermal decomposition: Stage I (room temperature to 200 °C), Stage II (200–600 °C), and Stage III (650–850 °C). These stages correspond to the release of volatiles, decomposition of organic matter and sulfates, and thermal decomposition of CaCO_3_, respectively. During Stage I, all samples show pronounced weight loss peaks. Notably, TSC-J shows a weight loss of approximately 1.41%, higher than 0.71% for TS and 0.79% for TSC, indicating higher volatile content at low temperatures. This is attributed to three factors: (1) EPS secreted during microbial solidification, which adsorb and retain capillary or weakly bound water [71]; (2) residual bound and adsorbed water from the CaCl_2_–urea binder; (3) thermal decomposition or volatilization of microbial residues and EPS (proteins, polysaccharides, etc.) between 30 and 300 °C [72]. In Stage II, TG curves remain relatively flat, indicating good thermal stability without significant decomposition. TSC-J shows slight, sustained weight loss, associated with gradual decomposition of organic components and amination products. During MICP, microbial cells and EPS adsorb Ca^2+^ to facilitate nucleation. Organics undergo dehydration, chain cleavage, and carbonization between 250 and 500 °C. Simultaneously, intermediate products from urea decomposition (e.g., NH_4_Cl, Ca(NH_2_COO)_2_) volatilize or decompose, slightly increasing weight loss. TS and TSC show minimal mass changes, confirming the superior structural stability of the titanium gypsum–clay system. Slight weight loss in Stage II mainly reflects residual curing materials, organic decomposition, and structural water release, rather than transformation of the primary mineral phase. Stage III shows a pronounced weight loss peak corresponding to CaCO_3_ decomposition (CaCO_3_ → CaO + CO_2_). TSC-J exhibits a weight loss of ~0.79% between 700 and 780 °C, higher than TS (0.27%) and TSC (0.39%), indicating microbial solidification promotes CaCO_3_ formation [73]. Calcite or aragonite formed via MICP decomposes between 650 and 800 °C. TSC-J shows a more pronounced peak and slightly lower decomposition temperature than pure CaCO_3_, likely due to finer grains, developed porosity, and matrix interactions [74]. Weight loss in this stage validates the effectiveness of microbial-induced CaCO_3_ precipitation and reveals transformation of carbonate phases into CaO. Compared to TS and TSC, higher weight loss in TSC-J at high temperatures confirms that microbial curing promotes mineral phase evolution while enhancing structural compactness and thermal stability.

#### 3.3.3. Microstructural Characteristics

**Figure 16:**
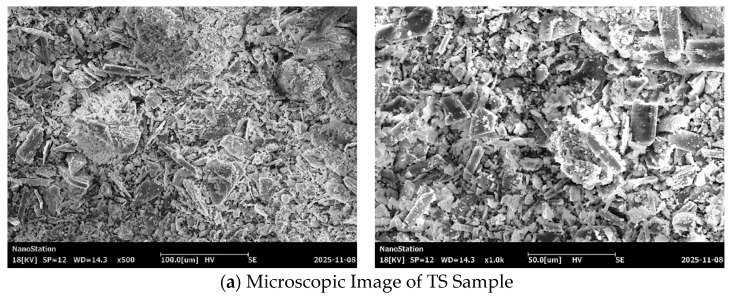

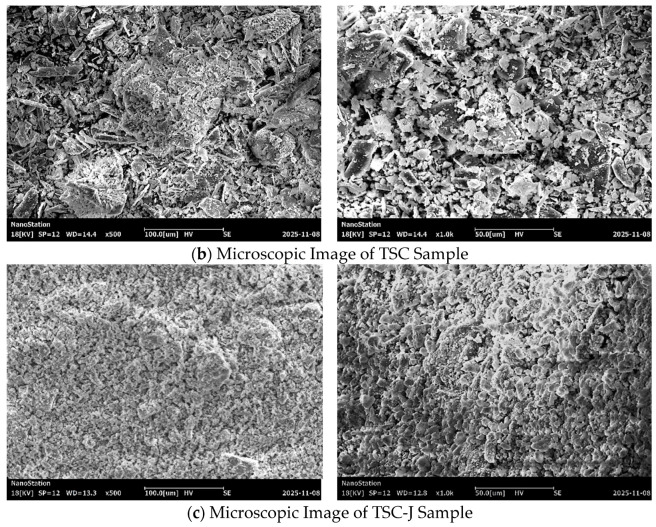
Comparison of TS, TSC, and TSC-J Samples at Different Magnifications.

To elucidate the regulatory mechanism of microbial mineralization on the microstructure of the TSC system and clarify the evolution of surface morphology, elemental composition, and pore structure, TS, TSC, and TSC-J samples were characterized using scanning electron microscopy (SEM), energy dispersive spectroscopy (EDS), and nitrogen adsorption–desorption analysis. This approach enables a micro-scale interpretation of the mechanisms underlying macro-performance enhancement. Figure 16 highlights significant differences in particle morphology, structural compactness, and interparticle bonding among the three sample groups [75]. The TS sample (Figure 16a) exhibits a loose surface structure, with crystals displaying typical plate-like and columnar morphologies arranged in a disordered pattern. Numerous inter-particle pores and gaps are present, characteristic of gypsum (CaSO_4_·2H_2_O) [63]. This indicates that TS predominantly consists of unmodified calcium sulfate crystals with weak internal cohesion and a loose overall structure, consistent with its low mechanical strength and water stability. In the TSC sample (Figure 16b), original gypsum plate-like crystals are partially covered or bonded, with a notable reduction in intergranular voids and increased structural density. Fine particles occupy spaces between gypsum crystals, forming a continuous matrix structure. This demonstrates that clay functions not only as a filler but also as a skeletal framework, optimizing particle grading and structural uniformity [76]. The TSC system exhibits clear micro-scale particle encapsulation and interlocking, providing favorable pore and interfacial environments for subsequent microbial-induced calcium carbonate precipitation. After Bacillus paracasei treatment, the TSC-J sample (Figure 16c) shows further microstructural densification. The surface is covered with abundant fine deposits, obscuring original particle boundaries and nearly filling all pores. Continuous cementation layers and agglomerated structures are evident, indicating the formation of new inorganic cementation within the system. This microstructural evolution enhances interparticle bonding and overall structural stability, directly correlating with the observed improvements in macroscopic mechanical performance and water stability of the TSC-J sample.

**Figure 17:**
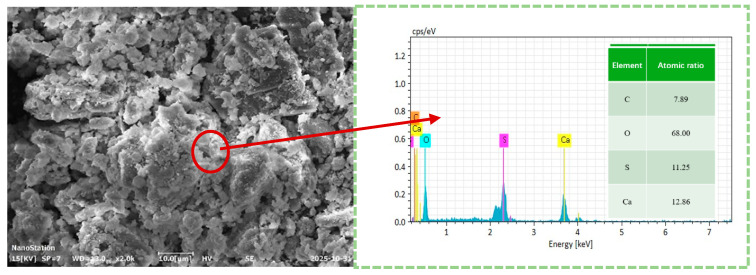
TSC-J Sample Point Scan Image.

To further elucidate the chemical composition of surface deposits on the TSC-J sample, energy-dispersive X-ray spectroscopy (EDS) point analysis was performed on representative regions, with the results presented in Figure 17. The analysis indicates that the area primarily comprises four elements: C, O, S, and Ca. Oxygen is dominant at 68.00 wt.%, followed by Ca (12.86 wt.%), S (11.25 wt.%), and C (7.89 wt.%). Combined with the preceding SEM observations, the high Ca and O contents suggest that these deposits predominantly consist of calcareous minerals. The EDS point-scan results are consistent with the area-scan data in Figure 12, confirming that microbially induced calcium carbonate (CaCO_3_) formation occurred within the TSC-J sample. Local enrichment of Ca and C on the sample surface indicates that the generated CaCO_3_ precipitates filled pores, enhanced interparticle cementation, and improved overall structural compactness. These microscale CaCO_3_ deposits [77] represent the core mechanism driving structural densification and enhanced water stability in the TSC system.

**Figure 18:**
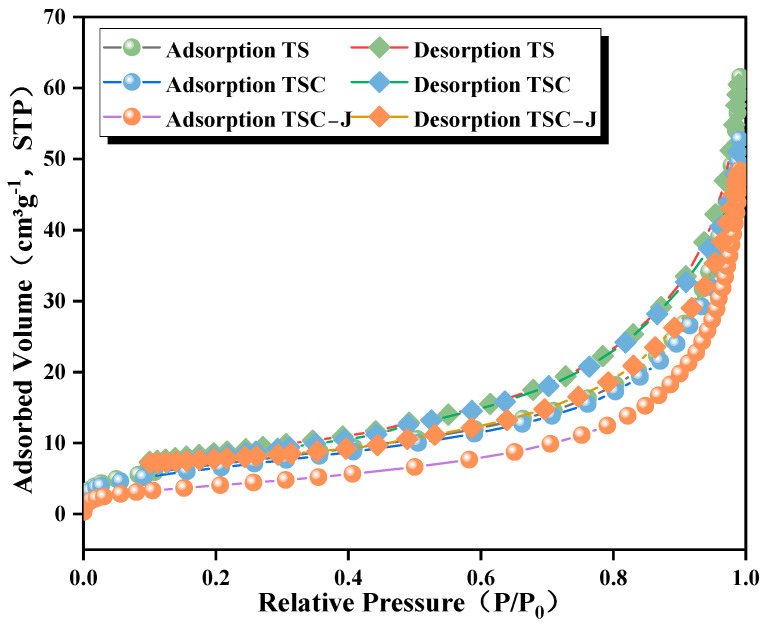
Nitrogen adsorption–desorption isotherms of TS, TSC, and TSC-J samples.

Figure 18 shows the nitrogen adsorption–desorption isotherms of TS, TSC, and TSC-J samples. All three samples exhibit typical Type IV isotherms with hysteresis loops in the relative pressure (P/P_0_) range of 0.4–0.9, indicating mesoporous structures. In the low-pressure region (P/P_0_ < 0.2), adsorption capacity is relatively low, corresponding to monolayer adsorption. As pressure increases, adsorption capacity rises sharply, accompanied by pronounced hysteresis loops, reflecting capillary condensation within the pores [78]. The TS sample shows the largest hysteresis loop and the highest adsorption capacity (~60 cm^3^·g^−1^), indicating well-developed pores. The TSC sample shows slightly lower values, suggesting partial pore filling caused by clay incorporation. The TSC-J sample exhibits the lowest adsorption capacity (~50 cm^3^·g^−1^) and the smallest hysteresis loop, indicating that CaCO_3_ precipitates formed during MICP curing fill mesopores and micropores, reducing overall pore volume and specific surface area, consistent with XRD analysis. Pore size distribution analysis indicates that all three samples are primarily concentrated within the 2–50 nm range. The TS sample exhibits a broad pore size distribution peaking around 20–25 nm. The TSC sample shows a reduced peak at 15–20 nm with a more uniform structure. The TSC-J sample displays a further narrowed distribution, with peaks at 10–15 nm and a significant decrease in macropores. This indicates effective CaCO_3_ filling both between particles and within pores, reducing pore connectivity and enhancing structural compactness [79]. In addition, the hysteresis loop observed in the relative pressure range of P/P_0_ ≈ 0.6–0.8 is consistent with capillary condensation behavior in mesoporous structures and is commonly reported in similar soil and cementitious materials. Compared with previous studies, this pressure range does not exhibit unusual characteristics but confirms that the pore system of the samples is dominated by mesopores. The relatively stable hysteresis behavior in this range further indicates that MICP treatment leads to a more uniform and refined pore structure, rather than introducing atypical pore distributions. In summary, nitrogen adsorption–desorption analysis demonstrates that MICP curing significantly enhances the pore structure of the TSC system, transforming it from highly porous to relatively compact. Consistent with SEM and XRD results, the generated CaCO_3_ acts as a “filling-binder” between particles, further confirming that MICP is the key microscopic mechanism enhancing the density and water stability of the cured samples.

### 3.4. Mechanism Diagram

**Figure 19:**
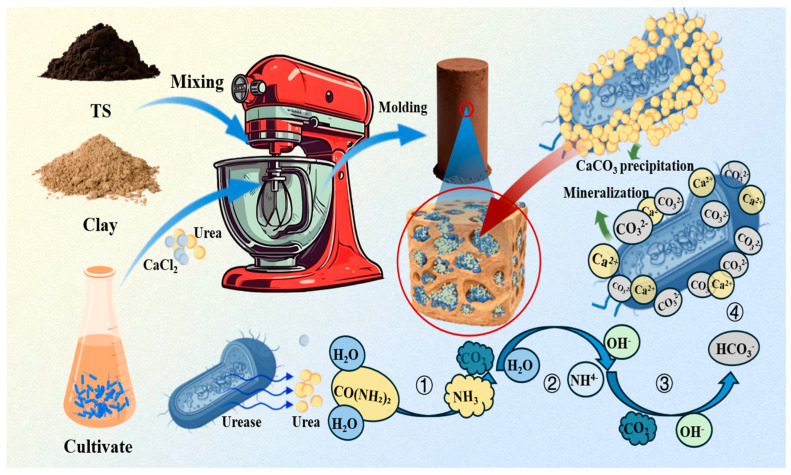
Schematic Diagram of Synergistic Modification Mechanism by Clay−Microbial Mineralization.

The mechanism of action in this study is systematically illustrated in Figure 19. First, titanium gypsum (TS) is mixed with clay at a specific ratio. Fine clay particles fill the large voids between TS particles, gradually converting the originally discrete particle size distribution into a continuous gradation. The composite mixture is then combined with a urease-active bacterial culture and a binder solution containing calcium chloride and urea, followed by molding. This process ensures uniform distribution of microorganisms, Ca^2+^ ions, and the solid matrix, providing reaction interfaces and diffusion pathways for mineralization. During subsequent reactions, Bacillus pseudomallei catalyzes the formation of calcium carbonate via urease. The complete mineralization process comprises four sequential stages (Figure 19): ① Urea hydrolyzes into NH_3_ and CO_2_ under urease action; ② NH_3_ reacts with water to form NH_4_^+^ and OH^−^, rapidly increasing system pH; ③ As the system becomes more alkaline, the CO_2_/HCO_3_^−^ equilibrium shifts toward CO_3_^2−^ accumulation; ④ CO_3_^2−^ preferentially nucleates and precipitates with Ca^2+^ on bacterial surfaces and within surrounding pores, ultimately forming CaCO_3_ crystals.

As mineralization progresses, CaCO_3_ gradually deposits as a cementing phase at particle contact points, pore throats, and framework surfaces, forming a characteristic “filling-cementation” reinforced structure. The deposited CaCO_3_ not only fills pores, reduces connectivity, and inhibits water erosion pathways but also forms a dense, continuous three-dimensional mineral cement network. Throughout this process, clay provides a uniform particle framework and adsorption sites, while microbially induced mineral deposition continuously remodels pores and strengthens cementation. Their synergistic action significantly improves water stability and long-term durability, enabling efficient utilization of TSC composite soil and substantial enhancement of its engineering performance.

## 4. Conclusions

This study investigates the stabilization behavior and water stability enhancement of a fine-grained composite soil system, referred to as TSC composite soil, characterized by weak fundamental properties and pronounced water sensitivity. A two-step modification strategy integrating clay blending regulation and microbial-induced mineralization was proposed, and the macroscopic performance evolution together with the underlying microstructural mechanisms were systematically examined. The main conclusions are summarized as follows.

(1) Clay blending enables targeted regulation of the fundamental physical properties of the TSC composite soil, with an optimal blending ratio of 40%. Under this condition, the particle size distribution is effectively optimized, and key engineering indices are significantly improved. Specifically, the liquid limit decreases from 67.82% to 37.84%, and the plasticity index is reduced by 44%, indicating a substantial mitigation of high plasticity and deformation susceptibility. Meanwhile, the California bearing ratio increases from 8.57% to 10.24%, the free expansion rate decreases from 42% to 27.2%, and the maximum dry density increases by 22.7%. These results indicate that clay blending can markedly improve the basic mechanical characteristics of the TSC composite soil; however, immersion tests indicate that the water stability coefficient remains relatively low (0.35–0.40), and the specimens are prone to disintegration, suggesting that particle structure regulation alone is insufficient to ensure adequate water resistance.

(2) Microbial-induced mineralization significantly enhances the water stability of the TSC composite soil through a multi-factor-regulated reinforcement process dominated by a filling–bonding mechanism. Using microbial-induced calcium carbonate precipitation (MICP) as the core strengthening approach, the optimal treatment conditions are identified as calcium chloride as the calcium source, a curing temperature of 30 °C, a system pH of 9, and a microbial solution dosage of 40%. Under these conditions, the 28-day water stability coefficient increases to 0.83, representing an improvement of approximately 83% compared with unmineralized specimens. These results confirm the effectiveness of microbial mineralization in enhancing the water resistance of fine-grained composite soils.

(3) Microstructural analyses reveal that microbially induced calcium carbonate effectively fills and coats the original plate-like sulfate minerals, leading to reduced pore volume and the formation of a continuous intergranular cementation network. FTIR results show a pronounced enhancement of carbonate-related absorption peaks accompanied by a weakening of sulfate-related signals, while XRD analysis indicates intensified calcite reflections, confirming the evolution toward a carbonate–sulfate composite mineral system. This pore-filling and interparticle bonding mechanism may underlie the observed improvements in mechanical strength and water stability of the TSC composite soil.

## Figures and Tables

**Table 1:** Main chemical composition of raw material.

Material	SO_3_	CaO	Fe_2_O_3_	SiO_2_	TiO_2_	Al_2_O_3_	Others
Titanium gypsum	35.5	31.2	8.5	3.9	2.4	2.7	15.8
Clay	-	-	1.15	62.36	-	22.48	14.01

**Table 2:** Basic physical properties of raw material.

Material	pH	Liquid Limit W_L_ (%)	Plastic Limit W_P_ (%)	Dry Density (kg·m^−3^)	Free Swelling Rate (%)
Titanium gypsum	9.46	67.82	30.96	1128.79	42
Clay	8.02	37.84	21.28	1582.1	26

**Table 3:** Compound test plan.

Test Number	TSC1	TSC2	TSC3	TSC4	TSC5
Titanium gypsum	90%	80%	70%	60%	50%
Clay	10%	20%	30%	40%	50%

## Data Availability

The original contributions presented in this study are included in the article. Further inquiries can be directed to the corresponding author.
